# Effects of host modulation through omega-3 dietary supplementation on inflammatory outcomes in periodontitis: a scoping review

**DOI:** 10.31744/einstein_journal/2024RW0936

**Published:** 2024-11-21

**Authors:** Aline Ramos Carlucci, Beatriz Rezende Bergo, Rafael Nascimento de Brito Silva, Gabriella de Deus Bressane, Mauricio Baeza, Nídia Castro dos Santos

**Affiliations:** 1 Universidade de São Paulo Department of Stomatology São Paulo SP Brazil Department of Stomatology, Universidade de São Paulo, São Paulo, SP, Brazil.; 2 Universidade Federal de Minas Gerais Belo Horizonte MG Brazil Universidade Federal de Minas Gerais, Belo Horizonte, MG, Brazil.; 3 Universidade de Guarulhos Dental Research Division Guarulhos SP Brazil Dental Research Division, Universidade de Guarulhos, Guarulhos, SP, Brazil.; 4 Universidade do Estado do Rio de Janeiro Department of Integrated Clinic Rio de Janeiro RJ Brazil Department of Integrated Clinic, Universidade do Estado do Rio de Janeiro, Rio de Janeiro, RJ, Brazil.; 5 University of Chile Center for Epidemiology and Surveillance of Oral Diseases Santiago Chile Center for Epidemiology and Surveillance of Oral Diseases, University of Chile, Santiago, Chile.; 6 Hospital Israelita Albert Einstein Faculdade Israelita de Ciências da Saúde Albert Einstein São Paulo SP Brazil Faculdade Israelita de Ciências da Saúde Albert Einstein, Hospital Israelita Albert Einstein, São Paulo, SP, Brazil.; 7 The Forsyth Institute The Forsyth Institute Cambridge MA United States The Forsyth Institute, The Forsyth Institute, Cambridge, MA, United States.

**Keywords:** Cytokines, Fatty acids, Inflammation, Omega-3, Periodontitis

## Abstract

**Objective:**

Inflammation causes the progressive destruction of the supporting tissues around teeth in patients with periodontitis. Therefore, this study aimed to investigate the immunological effects of omega-3 polyunsaturated fatty acids (n-3 PUFAs) as adjunctive therapy in patients with periodontal disease and identify potential biomarkers for the disease.

**Methods:**

This scoping review followed the Preferred Reporting Items for Systematic Reviews and Meta-Analyses guidelines to investigate the impact of omega-3 therapy with or without acetylsalicylic acid on the immunological parameters of periodontal treatment. Eligible studies included those conducted on patients with normoglycemia and diabetes, published after 2002 in English, and containing relevant keywords. The exclusion criteria included pre-2002 publications, literature reviews, animal studies, and articles without immunological analysis. This review involved careful study selection by two double-blind researchers using the Rayyan software, with data extraction and analysis performed by the third and fourth reviewers.

**Results:**

Seven randomized clinical trials that compared control/placebo and n-3 PUFA groups or the follow-ups of the n-3 PUFA groups were included. The concentration of inflammatory cytokines was reduced following dietary supplementation with n-3 PUFA in the reviewed studies. Specifically, IL-1β, TNF-α, IL-6, and RANKL levels were reduced after dietary supplementation with n-3 PUFA as an adjunctive therapy for periodontitis. Changes in inflammatory outcomes were associated with the clinical benefits of periodontitis. However, significant divergence in the evaluated inflammatory markers, samples, and methods impairs direct comparisons and quantitative analyses in the available literature.

**Conclusion:**

This study highlights the need for clinical trials to advance our understanding and assessment of inflammatory outcomes in patients with periodontitis.

## INTRODUCTION

Periodontitis is a chronic inflammatory disease initiated by dysbiotic biofilms. Inflammation is a disease marker that leads to the progressive destruction of the supporting tissues around teeth, potentially resulting in tooth mobility and loss.^(1–3)^ Subgingival instrumentation is the standard treatment for periodontal disease because it mechanically disrupts and removes biofilm-derived pathogens.^(4)^ However, some patients with periodontal disease do not respond well to this treatment. This highlights the need for additional therapies to promote periodontal and systemic health.^(5)^

The predisposition of the host must be considered as an inseparable factor for both periodontitis pathogenesis and control to improve periodontal health. Individuals with this disease exhibit a hyperreactive immunoinflammatory profile and have hyperfunctional neutrophils, which contribute to the increased release of pro-inflammatory mediators.^(6,7)^ Moreover, tissue destruction provides an ecological niche conducive to dysbiosis by promoting the presence of periodontal pathogens.^(6)^ Therefore, immunological factors and lifestyle changes such as a balanced diet, probiotic supplementation, and good oral hygiene should be considered for the successful control and prevention of periodontal disease.^(6)^ Thus, supplementation with omega-3 polyunsaturated fatty acids (n-3 PUFAs), with or without acetylsalicylic acid (ASA), has been suggested as an adjunctive therapy that can modulate the host response to reverse the exacerbated inflammation associated with the disease.^(8,9)^

Omega-3 PUFAs are found in various foods and have potential health benefits. Their modulatory capacity has been observed in clinical studies of different inflammatory conditions such as cardiovascular disease and diabetes.^(10–13)^ When fish oil is consumed, eicosatetraenoic acid (EPA) and docosahexaenoic acid (DHA) promote the release of specialized pro-resolving mediators such as resolvins, maresins, and proteins.^(8,10)^ These mediators play multiple roles in the resolution of inflammation, including the inhibition of leukocyte recruitment and neutrophil infiltration, modulation of cytokine synthesis, and macrophage-induced stimulation of phagocytosis.^(8–10,14,15)^ Low-dose aspirin ASA is occasionally included within certain protocols, owing to the interaction between aspirin and the metabolism of EPA and DHA. This interaction leads to the generation of additional potent pro-resolving mediators^(16)^ that restore tissue homeostasis and initiate the repair phase. Human studies have highlighted the correlation between different host modulation methods and their respective contributions to improvements in clinical parameters in patients with periodontitis, such as attachment level gain and probing pocket depth reduction.^(17–19)^ However, the effects of n-3 dietary supplementation on inflammatory outcomes have not been critically reviewed.

**Figure 1:**
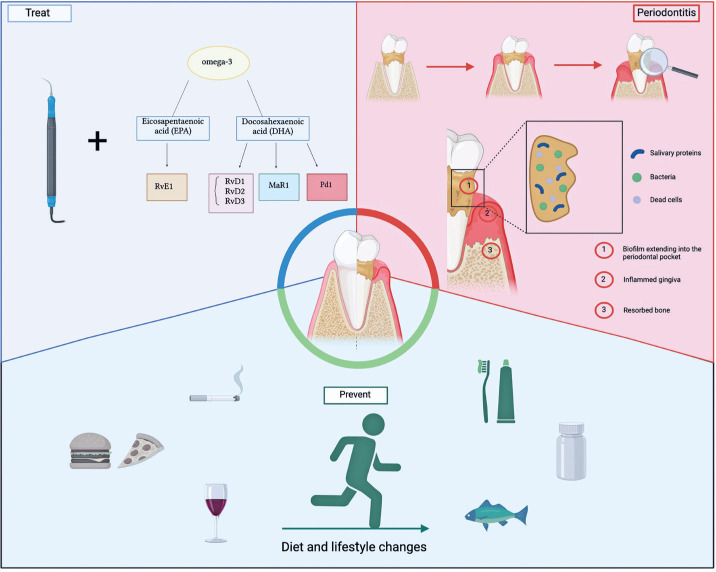
Illustration of the pathogenesis, prevention, and treatment of periodontitis

Inflammatory mediators can indicate the severity and progression of periodontal disease.^(20)^ Increased levels of inflammatory markers such as cytokines and prostaglandins reflect the immune response and extent of tissue damage.^(6,7)^ Therefore, this scoping review comprehensively screened current evidence from human trials and discusses the significance and implications of inflammatory outcomes following n-3 PUFA dietary supplementation in patients with periodontitis. This review further discusses possible future applications of the current knowledge to unravel the modulatory effects of n-3 PUFA in humans (Figure 1).

## METHODS

### Protocol

We performed systematic research in accordance with the Preferred Reporting Items for Systematic Reviews and Meta-Analyses (PRISMA-ScR) guidelines for Scoping Reviews (https://osf.io/m57xc).^(21)^ The search was conducted in March 2023 by two independent and calibrated reviewers (ARC and BRB) using the Rayyan software (Qatar Computing Research Institute, Doha, Qatar) to select the paper filters in digital databases.^(22)^

### Focused question

This review sought to answer the following question: "How does adjunctive n-3 therapy affect inflammatory outcomes in patients with periodontitis?"

### Type of studies and participants

The inclusion criteria were as follows: Studies in patients with normoglycemia and *diabetes mellitus*; randomized or non-randomized controlled clinical trials; observational studies (analytical or descriptive); studies with at least two keywords in their titles and abstracts; and studies that collected biological samples to evaluate the inflammatory outcomes of n-3 in periodontitis. No restrictions on age or number of patients were considered.

### Exclusion criteria

The following exclusion criteria were applied: Letters to the editor; literature reviews; case series; case-control studies; conference abstracts and editorials; *in vitro* studies; as well as studies that used animal models, did not demonstrate immunological analysis, did not collect biological samples for immunological analysis, and included smokers or patients without periodontal disease.

### Interventions and comparisons

The included studies had to promote protocols of periodontal care in which patients would receive oral hygiene guidance; supra-gingival scaling; as well as referrals for other necessary dental treatments (endodontics, restorations, extractions), scaling and root planing (SRP), and host modulation therapy based on n-3 PUFA with or without the addition of ASA. Comparisons are analyzed in the Results section, addressing similarities and differences between the dosages used, whether administration occurred before or after SRP, the type of biological material collected, and how the changes in inflammatory markers were assessed during the treatment period.

### Outcome measures

The included studies reported the results of immunological analyses to assess the inflammatory outcomes of the adjunctive use of n-3 PUFA. These results were expected to include the analysis of signaling proteins (cytokines, soluble cytokines, chemokines, and growth factors) with both pro- and anti-inflammatory characteristics at baseline and subsequent time points during treatment follow-up. The reviewed literature further reported all steps of sample collection, storage, laboratory analytical techniques, and statistical analyses.

### Information sources

Several prominent digital databases known for their extensive collection of medical and scientific literature were selected. These were the National Library of Medicine MEDLINE/PubMed, Embase, BVSalud, Web of Science, and Cochrane Library.

### Search strategy

A comprehensive search strategy was developed to identify relevant studies on the relationship between periodontitis and n-3 PUFA. This strategy was tailored to each digital database to ensure inclusion of all pertinent articles covering a broad spectrum of research. Furthermore, filters for titles, abstracts, and keywords were applied to all databases.

The keywords used for MEDLINE/PubMed, Web of Science, and BVSalud were: "periodontitis" OR "periodontal disease" AND "polyunsaturated fatty acids omega 3" OR "ω-3" OR "omega 3 therapy".

The search in the Embase database incorporated Emtree terms and free text to cover the terminology used in the different studies. The following keywords were used: "periodontitis" OR "periodontal disease" AND "omega 3 therapy". The keywords used for the Cochrane Library were: "periodontitis" OR "periodontal disease" AND "polyunsaturated fatty acids omega 3" OR "ω-3" OR "omega 3 therapy".

The search focused on systematic reviews and clinical trials to ensure the inclusion of high-quality evidence. However, no articles from this database were used because only systematic reviews were found.

Adapting the search strategy for each database enabled the retrieval of relevant studies, ensuring a comprehensive review of the role of omega-3 fatty acids in patients with periodontal disease.

### Study selection

Two double-blind researchers (ARC and BRB) performed all the literature search steps using Rayyan software. The full-text papers were read by a third reviewer to confirm whether the selected studies met the inclusion criteria. Any persistent disagreements regarding inclusion were resolved by a third reviewer (RNBS). All included studies were assessed, and quantitative data analysis was performed. Subsequently, a fourth reviewer (GDB) manually evaluated all references in each selected study to extract data and identify additional data. For data extraction, the data were divided into demographic data, the allocation of patients to the test groups of each survey, the region where the surveys were conducted, and the therapeutic approach used.

## RESULTS

In total, 5,088 articles published between 2002 and 2024 were identified, evaluated, and selected based on the inclusion criteria. The Rayyan software identified 3,466 duplicate articles that were subsequently excluded. In addition, 1,622 articles were selected for reading titles and excluded according to the selection criteria.

**Figure 2:**
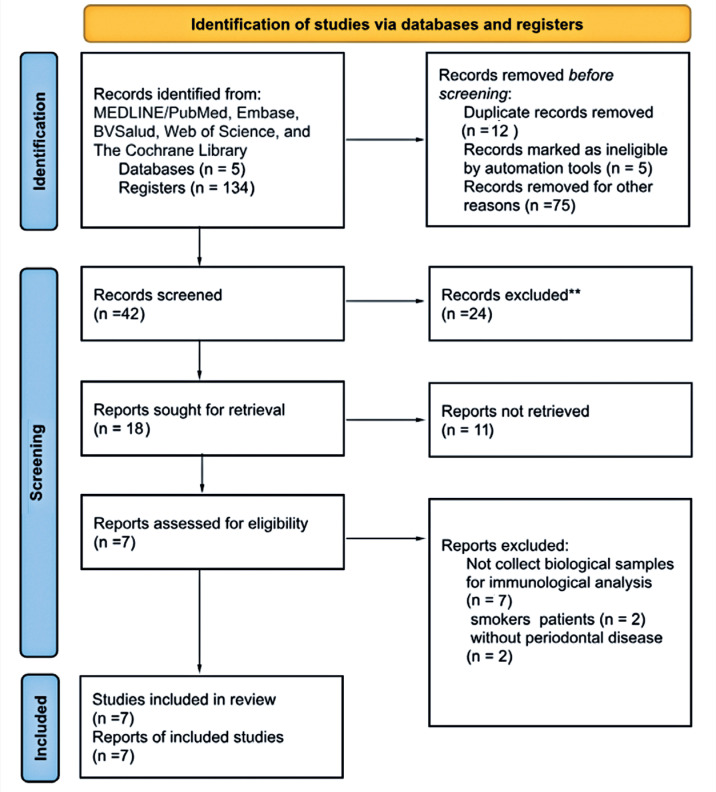
The study selection process

Initially, 134 articles were found in the databases, and 12 duplicate papers were excluded. For the first selection based on the title, 122 papers were analyzed, resulting in 42 selected papers. Next, 42 abstracts were read, and 24 were excluded (reasons). Finally, 18 full texts were carefully read and analyzed for eligibility (inclusion/exclusion), totaling seven papers analyzed in this review. The search strategy is illustrated in figure 2.

The reviewed literature included seven placebo-controlled randomized clinical trials (RCTs). In total, 353 participants were included: 169 and 194 in the control and omega-3 test groups, respectively. The SRP was performed in both the test and control groups (Table 1).

**Table 1:** Population data of participants included in each study, regions of performance, information on therapy and medication dosage, and description of the methodology for collecting immunological analysis material for each included study

	Authors and year														
	El-Sharkawy et al. ^(23)^ 2010		Elwakeel et al. ^(24)^ 2015		Keskiner et al. ^(25)^ 2017		Umrania et al. ^(26)^ 2017		Stańdo et al. ^(27)^ 2020		Castro dos Santos et al. ^(28)^ 2020			Deore et al. ^(29)^ 2014	
Location	Egypt		Egypt		Turkey		India		Poland		Brazil			India	
Study type	Placebo-controlled, double-blind, RCT		Placebo-controlled, double-blind RCT		Placebo-controlled, double-blind, RCT		RCT		RCT		Placebo-controlled, double-blind RCT			Placebo-controlled, double-blind, RCT	
*Diabetes mellitus*	No		Yes		No		No		No		Yes			No	
**Groups**	**Control**	**Test**	**Control**	**Test**	**Control**	**Test**	**Control**	**Test**	**Control**	**Test**	**Control**	**Test 1**	**Test 2**	**Control**	**Test**
Number of patients	40	40	20	20	15	15	20	20	20	20	25	25	25	29	29
Age	44.2 ± 8.4	46.1 ± 7.6	40.05 ± 9	40.05 ± 9	40.87 ± 9.7	42.54 ± 5.82	43.5 ± 5.8	44 ± 6.44	54 ± 11	47.5 ± 9.63	54.9 ± 9.7	55.6 ± 8.3	54.4 ± 10.2	44.47 ± 5.2	45.40 ± 40.90
Gender	not described	not described	10 Male - 10 Female	10 Male - 10 Female	8 Male - 7 Female	8 Male -7 Female	13 Male - 7 Female	13 Male - 7 Female	9 Male - 11 Female	10 Male - 10 Female	9 Male - 16 Female	9 Male - 16 Female	13 Male -12 Female	not described	not described
Intervention	SRP + placebo	SRP + 3 g ω-3 + 81 mg ASA / 3× daily (6 months)	SRP + placebo	SRP + 3 g ω-3 + 75 mg ASA / 3× daily (6 months)	SRP + placebo	SRP + 6.25 mg EPA + 19.19 mg DHA / 2× daily (6 months)	SRP	SRP + 700 mg ω-3 / 1× daily (3 months)	SRP	SRP + 10 mL ω-3 /2× daily (3 months)	Debridement + placebo	3 g ω-3 [3× daily] + 100 mg ASA [1× daily] (2 months) AFTER SRP	3 g ω-3 [3× daily] + 100 mg ASA [1× daily] (2 months) BEFORE SRP	SRP + placebo	SRP + 300 mg ω-3 PUFA orally ingested as one capsule daily for 12 weeks
PUFA	900 mg		900 mg		214 mg		700 mg		not described		900 mg			300 mg	
EPA (mg/%)	13%		13%		6,25 mg		180 mg		2600 mg		540 mg			180 mg	
DHA (mg/%)	9%		9%		19,19 mg		120 mg		1800 mg		360 mg			120 mg	
Composition and dose of Placebo	not described		ω-3: coconut oil; ASA: lactose tablet		not described		no placebo		no placebo		not described			not described	
Type of sample and amount collected	5 mL unstimulated saliva		GCF		5 mL unstimulated saliva		unstimulated saliva		5 mL unstimulated saliva		GCF			Blood	
Conditioning	Saliva was collected in an empty tube in the morning, stored at 80 °C, and centrifuged at 10,000× *g* for 5 min; the supernatants were collected.		For each marker, a Periopaper strip was inserted into the pocket for 30 s. Special attention was given to prevent mechanical trauma.		Saliva was collected in an empty tube in the morning, stored at 80 °C, and centrifuged at 10,000 × *g* for 10 min at 4 °C; the supernatants were then collected.		Saliva samples were collected through expectoration into polypropylene tubes and stored at -20 °C.		Saliva was collected in a 50 mL Falcon tube before clinical measurements and centrifuged at 10,000 × *g* for 10 min at 4 °C. The supernatants were subsequently collected in 1.5 mL tubes and frozen at -80 °C.		GCF was collected using Periopaper strips, which were inserted into the pockets until a slight resistance was felt and kept there for 30 s. The samples were placed in sterile microtubes and kept frozen until the Multiplex assay.			Not described	
Analysis method	ELISA		ELISA		ELISA		ELISA		Multiplex ELISA		Multiplex ELISA			Not described	
Collection times	Baseline, 3 mo, and 6 mo		Baseline, 3 mo, and 6 mo		Baseline, 1 mo, 3 mo, and 6 mo		Baseline and 3 mo		Baseline and 3 mo		Baseline, 3 mo, and 6 mo			Baseline, 6 weeks, and 12 weeks	
Analyzed immunological parameters	RANKL (pg/mL), MMP-8 (ng/mL)		IL-1β (pg/mL), MCP-3 (pg/mL)		TNF-α (pg/mL), SOD (U/mL)		IL-1β		IL-1β, IL-1RA, IL-2, IL4, IL-5, IL-6, IL-7, IL-9, IL-10, IL-12, IL-13, IL-15, IL-16, IL- 17, IFN-γ, MIF, TNF-α, CCL1/I-309, CCL2/MCP-1, CCL3/MIP-1α, CCL4/MIP- 1β, CCL5/RANTES, CCL7/MCP-3, CCL8/MCP-2, CCL11/Eotaxin, CCL13/MCP-4, CCL15/MIP-1delta, CCL17/TARC, CCL19/MIP-3β, CCL20/MIP-3α, CCL21/6Ckine, CCL22/MDC, CCL23/MPIF-1, CCL24/Eotaxin-2, CCL25/TECK, CCL26/Eotaxin-3, CCL27/CTACK, CX3CL1/Factalkine, CXCL1/Gro-alpha, CXCL2/Gro-beta, CXCL5/ENA-78, CXCL6/GCP-2, CXCL8/IL-8, CXCL9/MIG, CXCL10/IP-10, CXCL11/I-TAC, CXCL12/SDF-1α, CXCL13/BCA-1, CXCL16/SCYB16, FGF2, G-CSF, GM-CSF, PDGF-BB abd VEGF (all pg/mL)		IL-1β, TNF-α, IL-6, IL-8, MIP-1α, MCP-1, IFN-γ, IL-4, IL-10 (all pg/mL)			CRP (mg/mL)	

RCT: randomized clinical trial; GCF: gingival crevicular fluid; SRP: scaling and root planing; ASA: acetylsalicylic acid; ω-3: omega-3; ELISA: enzyme-linked immunosorbent assay; Mo: months; M: male; F: female; EPA: eicosatetraenoic acid; DHA: docosahexaenoic acid; PUFA: polyunsaturated fatty acid.

Participants in the assessed studies had an average age of 46.76 ± 10.83 years, and gender distribution was reported in five studies. The duration of n-3 supplementation varied, with three studies employing a 6-month regimen, three utilizing a 3-month protocol, and only one opting for a 2-month regimen. Moreover, four studies detailed the daily intakes of EPA and DHA, which ranged from 180mg to 2.6g (Table 1). Furthermore, three studies incorporated a daily intake of 900mg PUFA. Immunological biomarkers were scrutinized, revealing a reduction in IL-1β, TNF-α, RANKL, and IL-6 levels between baseline and different follow-ups.

Sample preparation methods differed among the selected studies. Samples were collected using non-stimulated saliva, stored at -80 °C, and centrifuged for 5 min in three studies. Two studies involving gingival crevicular fluid (GCF) samples utilized various methods, such as the collection of fluid with Periopaper strips and conducting a multiplex assay, with samples stored in a frozen state. However, the studies involving blood samples did not provide detailed information regarding the procedure. Moreover, enzyme-linked immunosorbent assay (ELISA) was the predominant analysis method, except in one study. The samples analyzed included saliva, GCF, and serum (Table 1).

## DISCUSSION

**Figure 3:**
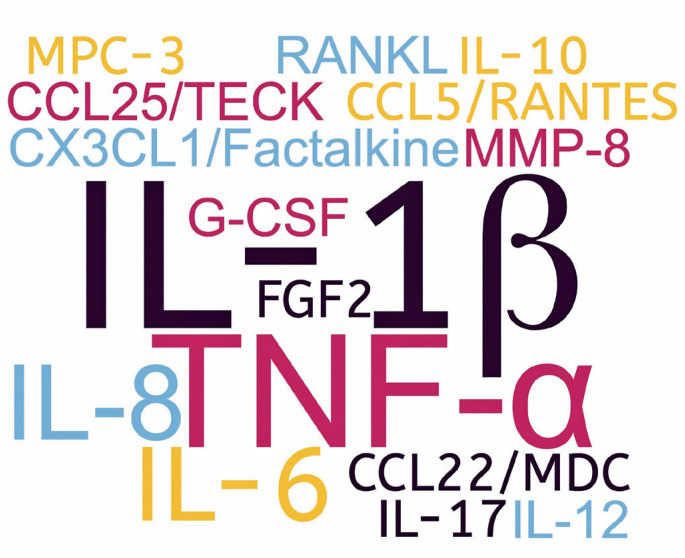
Immune parameters more frequently affected by n-3 PUFA supplementation as an additional treatment in the reviewed studies. IL-Iβ levels were altered in two studies, whereas the remaining parameters were each altered in only one study

Dietary supplementation with n-3 PUFA as an adjunctive therapy for periodontitis changed inflammatory outcomes in humans. The most prevalent inflammatory outcomes assessed in the studies were IL-1β and TNF-α levels, which are key inflammatory cytokines. Adjunctive dietary supplementation with n-3 PUFA reduced the concentrations of IL-1β, TNF-α, IL-6, and RANKL in saliva, GCF, or serum among patients with periodontitis (Figure 3).^(23–28)^

Collection of GCF was more consistent between the two studies that reported altered IL-Iβ levels.^(28,29)^However, only one study provided details on the storage of samples until analysis.^(29)^ Of the studies that examined saliva, two specified the collection time.^(23,25)^ Saliva samples were refrigerated in all studies, with three studies storing the samples at -80°C^(23,25,27)^ and one at -20°C.^(26)^ Additionally, all samples were centrifuged before analysis, with the duration of this process varying for supernatant separation.^(23,25,27)^ However, the only study that analyzed blood did not provide details on how the samples were collected, stored, and processed.^(29)^

The modulatory effects of n-3 PUFA dietary supplementation on periodontitis are associated with a reduction in proinflammatory cytokines, resulting in diminished tissue degradation and decreased substrate provision for periodontal pathogens.^(30,31)^ Moreover, n-3 PUFA supplementation promotes the restoration of immune response balance in the host.^(32)^ These mediators contribute to the pro-resolution phase of the inflammatory process by activating non-phagocytic macrophages that are vital for tissue homeostasis and regeneration.^(33,34)^

IL-1β was the most prevalent inflammatory marker in human studies. Dietary supplementation with n-3 PUFA in periodontal treatment reduced the concentration levels of IL-1β in saliva and GCF, indicating a host modulatory effect of this adjunctive therapy. This family of pro-inflammatory cytokines contributes to the initiation and progression of periodontal disease by promoting leukocyte migration and recruitment, stimulating the production of other inflammatory mediators and metalloproteinases, activating T and B lymphocytes, and stimulating osteoblasts, leading to bone resorption.^(30,31)^ DHA and EPA n-3 PUFA modulate the inflammatory cascade by effectively inhibiting the release of new pro-inflammatory mediators such as IL-1β. These findings highlight the beneficial anti-inflammatory effects of DHA and EPA and indicate that n-3 is a promising intervention for managing periodontal inflammatory conditions.^(23–28)^

TNF-α plays a major role in the architecture of aberrant immune response through various functions. These include the positive induction of adhesion molecules, favoring cell migration. In addition, it facilitates extracellular matrix degradation and bone resorption by promoting the secretion of matrix metalloproteinases and RANKL, as well as stimulating the production of chemokines.^(30)^ In this scoping review, we observed a reduction in TNF-α salivary levels in the groups receiving n-3 compared to the control groups. These findings demonstrate the beneficial effect of n-3 PUFA dietary supplementation in individuals with periodontitis.^(25,27)^

In periodontal disease, bone resorption occurs because of increased osteoclastic activity mediated by RANKL, the principal regulator of osteoclast precursor cell differentiation. RANKL is secreted as a soluble protein by osteoblasts, fibroblasts, bone marrow cells, and T cells in response to pro-inflammatory cytokines such as TNF-α and IL-1.^(35)^ Salivary levels of RANKL were lower in patients who received n-3 supplementation than in those who underwent only scaling and root planing.^(23)^ This suggests that the modulatory effects of n-3 PUFA could be extended to bone homeostasis, owing to their ability to promote osteoblastogenesis by suppressing the expression of specific osteoclast genes and inhibiting osteoblast migration and adhesion.^(35)^

The assessed studies also demonstrated a reduction in IL-6 levels. This cytokine is synthesized and released by various cells, including endothelial cells, fibroblasts, neutrophils, monocytes, and macrophages. Elevated IL-6 levels are frequently observed in patients with chronic inflammatory diseases. Thus, they are considered a biological marker of diabetic complications and the risk of mortality among patients with diabetes and cardiovascular diseases.^(36)^ IL-6 mediates the recruitment and activation of immune cells, particularly neutrophils. This leads to an intensified inflammatory response within the gingival tissues. This exacerbated immune reaction results in the degradation of periodontal tissues and the subsequent loss of bone and connective tissue support around the teeth. Moreover, IL-6 disrupts the equilibrium between pro- and anti-inflammatory responses, further exacerbating the destructive nature of periodontal disease.^(36)^

Dysregulation of IL-6 in periodontitis not only perpetuates the inflammatory process but also contributes to impaired tissue repair and regeneration, hindering the natural healing mechanisms in the periodontium.^(36)^ The GCF levels of this cytokine increase in patients with periodontal disease and decrease after periodontal treatment.^(36,37)^ One study demonstrated that the GCF levels of IL-6 were lower in patients who received n-3 supplementation than in those who underwent periodontal debridement alone.^(28)^ In contrast, another study revealed no significant differences in IL-6 levels in saliva samples between the test and control groups.^(27)^

Biological markers play pivotal roles in translational research and personalized medicine. They identify individual characteristics, predict treatment responses, and demonstrate disease progression.^(38)^ These markers bridge the gap between scientific advancement and clinical practice, facilitating the development of tailored therapies based on solid evidence. Therefore, their identification aids in customizing treatments and enhancing clinical outcomes in oral health.^(39)^ In addition, the use of biological markers provides a solid foundation for clinical decision-making.^(40)^ The identification of biological markers of periodontitis, such as inflammatory cytokines, can provide valuable insights into patient responses to treatment and disease progression. These markers can help in personalized therapy and improvement of clinical outcomes.^(41–43)^ Therefore, future studies should target translational analyses based on previous findings reported in the literature, allowing qualitative and quantitative comparisons to substantiate current scientific advances. Selection of inflammatory markers and standardization of sampling processes and analyses can provide evidence regarding biological outcomes in clinical practice and personalized medicine.

The development of omics has rendered advancements in biomedical analyses more profound and precise.^(44)^ In periodontitis, approaches such as proteomics and transcriptomics may help identify inflammatory biomarkers and elucidate disease pathogenesis. Furthermore, these techniques can aid in the validation of treatments and implementation of personalized periodontal therapies.^(45)^ Metabolomic techniques would enable the identification of specific bioactive mediators and mapping of their biosynthetic pathways to directly assess the effects of n-3 administration on the immune response.^(46)^ Despite their cost and complexity, these tools can help determine whether alterations in inflammatory levels can be attributed to n-3 PUFA. This is crucial because the exclusive analysis of proteinaceous inflammatory mediators limits such assertions, underscoring the benefits of analyzing lipid mediators and their physiological pathways in future studies.^(46)^

Based on these findings, this scoping review presents a perspective on the scientific literature in this domain. It examines the correlation between adjunctive n-3 therapy for periodontitis and discernible improvements in inflammatory outcomes in humans. Elucidating the mechanisms by which n-3 PUFA can resolve inflammation in humans will significantly contribute to scientific advancement, guiding future translational studies to clarify the biological foundations of periodontal therapy and its relevance in clinical practice. Furthermore, the implementation of a comprehensive protocol that encompasses the assessment of inflammatory outcomes and addresses practical considerations for sample collection can markedly advance our understanding of host modulation therapies in periodontal care. By considering the immune-inflammatory aspects of treatment outcomes, we can acquire valuable insights into the mechanisms of action and efficacy of adjunctive therapies for periodontitis, such as n-3 PUFA dietary supplementation. Integration of the assessment of inflammatory outcomes into clinical trial protocols provides a more comprehensive approach to periodontal treatment. This enhances our ability to assess the influence of these therapies in resolving inflammation in humans.

It is also important to understand the role of n-3 PUFA in modulating the immune response because lipid-derived mediators can resolve inflammation and promote tissue repair. These processes are essential for periodontal health and are often compromised in patients with diabetes. The inclusion of both patients with diabetes and individuals with normal glycemia, especially considering the influence of diabetes on the inflammatory response, elucidated the effects of n-3 PUFA supplementation in different periodontal health contexts. This comprehensive approach allowed a more robust analysis of the potential benefits of n-3 PUFA to the inflammatory response in patients with various health conditions. Therefore, our findings and interpretations contribute to a more complete understanding of the impact of the proposed adjunct therapy in patients with periodontitis.

One limitation of this study is the use of diverse biological samples, including saliva, GCF, and serum, to analyze inflammatory markers. The heterogeneity observed among clinical studies poses an obstacle to establishing quantitative comparisons between studies and their respective outcomes. Moreover, the lack of standardization in the administration of n-3 PUFA as an adjunct to non-surgical periodontal treatment, both in terms of administration methods and dosages employed in clinical trials, impaired direct comparisons between clinical studies. Despite these limitations, this study highlights the inflammatory repercussions of dietary supplementation with n-3 PUFA and their importance. This demonstrates the beneficial modulatory effects of the adjunctive use of n-3 PUFA to treat periodontitis in rebalancing the host immune response.^(47)^ Despite these promising indications, the widespread implementation of this therapy requires more substantial support through additional RCTs. In addition, further investigations are required to validate and deepen our understanding of the potential benefits of this therapeutic approach.

## CONCLUSION

The reduction of IL-1β, TNF-α, IL-6, and RANKL levels after dietary supplementation with n-3 PUFA as an adjunctive therapy for periodontitis was the most prevalent among the reviewed studies. The available evidence demonstrates that new human clinical trials evaluating the effects of dietary supplementation with n-3 PUFA in the treatment of periodontitis should include the assessment of inflammatory outcomes. Thus, this review identifies opportunities for additional advancements in this field.

## References

[1] Cochran DL (2008). Inflammation and bone loss in periodontal disease. J Periodontol.

[2] Institute for Health Metrics and Evaluation (IHME) (2020). Global Burden of Disease Study 2019 (GBD 2019) Data Resources.

[3] Hajishengallis G, Chavakis T (2021). Local and systemic mechanisms linking periodontal disease and inflammatory comorbidities. Nat Rev Immunol.

[4] Sanz M, Herrera D, Kebschull M, Chapple I, Jepsen S, Beglundh T (2020). EFP Workshop Participants and Methodological Consultants. Treatment of stage I-III periodontitis-The EFP S3 level clinical practice guideline. J Clin Periodontol.

[5] Tomasi C, Leyland AH, Wennström JL (2007). Factors influencing the outcome of non-surgical periodontal treatment: a multilevel approach. J Clin Periodontol.

[6] Loos BG, Van Dyke TE (2020). The role of inflammation and genetics in periodontal disease. Periodontol 2000.

[7] Hajishengallis G (2014). Immunomicrobial pathogenesis of periodontitis: keystones, pathobionts, and host response. Trends Immunol.

[8] Hajishengallis G, Chavakis T, Lambris JD (2020). Current understanding of periodontal disease pathogenesis and targets for host-modulation therapy. Periodontol 2000.

[9] Balta MG, Papathanasiou E, Blix IJ, Van Dyke TE (2021). Host Modulation and Treatment of Periodontal Disease. J Dent Res.

[10] Calder PC (2010). Omega-3 fatty acids and inflammatory processes. Nutrients.

[11] Hu Y, Hu FB, Manson JE (2019). Marine Omega-3 Supplementation and Cardiovascular Disease: An Updated Meta-Analysis of 13 Randomized Controlled Trials Involving 127 477 Participants. J Am Heart Assoc.

[12] Ajabnoor SM, Thorpe G, Abdelhamid A, Hooper L (2021). Long-term effects of increasing omega-3, omega-6 and total polyunsaturated fats on inflammatory bowel disease and markers of inflammation: a systematic review and meta-analysis of randomized controlled trials. Eur J Nutr.

[13] Simopoulos AP (2002). Omega-3 fatty acids in inflammation and autoimmune diseases. J Am Coll Nutr.

[14] Thornton JM, Yin K (2021). Role of Specialized Pro-Resolving Mediators in Modifying Host Defense and Decreasing Bacterial Virulence. Molecules.

[15] Eltay EG, Van Dyke T (2023). Resolution of inflammation in oral diseases. Pharmacol Ther.

[16] Spite M, Serhan CN (2010). Novel lipid mediators promote resolution of acute inflammation: impact of aspirin and statins. Circ Res.

[17] Castro dos Santos NC, Furukawa MV, Oliveira-Cardoso I, Cortelli JR, Feres M, Van Dyke T (2022). Does the use of omega-3 fatty acids as an adjunct to non-surgical periodontal therapy provide additional benefits in the treatment of periodontitis? A systematic review and meta-analysis. J Periodontal Res.

[18] Heo H, Bae JH, Amano A, Park T, Choi YH (2022). Supplemental or dietary intake of omega-3 fatty acids for the treatment of periodontitis: a meta-analysis. J Clin Periodontol.

[19] Van Ravensteijn MM, Timmerman MF, Brouwer EA, Slot DE (2022). The effect of omega-3 fatty acids on active periodontal therapy: A systematic review and meta-analysis. J Clin Periodontol.

[20] Kalsi AS, Moreno F, Petridis H (2021). Biomarkers associated with periodontitis and peri-implantitis: a systematic review. J Periodontal Implant Sci.

[21] Tricco AC, Lillie E, Zarin W, O’Brien KK, Colquhoun H, Levac D (2018). PRISMA Extension for Scoping Reviews (PRISMA-ScR): Checklist and Explanation. Ann Intern Med.

[22] Ouzzani M, Hammady H, Fedorowicz Z, Elmagarmid A (2016). Rayyan-a web and mobile app for systematic reviews. Syst Rev.

[23] El-Sharkawy H, Aboelsaad N, Eliwa M, Darweesh M, Alshahat M, Kantarci A (2010). Adjunctive treatment of chronic periodontitis with daily dietary supplementation with omega-3 Fatty acids and low-dose aspirin. J Periodontol.

[24] Elwakeel NM, Hazaa HH (2015). Effect of omega 3 fatty acids plus low-dose aspirin on both clinical and biochemical profiles of patients with chronic periodontitis and type 2 diabetes: a randomized double-blind placebo-controlled study. J Periodontal Res.

[25] Keskiner I, Saygun I, Bal V, Serdar M, Kantarci A (2017). Dietary supplementation with low-dose omega-3 fatty acids reduces salivary tumor necrosis factor-α levels in patients with chronic periodontitis: a randomized controlled clinical study. J Periodontal Res.

[26] Umrania VV, Rao Deepika PC, Kulkarni M (2017). Evaluation of dietary supplementation of omega-3 polyunsaturated fatty acids as an adjunct to scaling and root planing on salivary interleukin-1β levels in patients with chronic periodontitis: a clinico-immunological study. J Indian Soc Periodontol.

[27] Stańdo M, Piatek P, Namiecinska M, Lewkowicz P, Lewkowicz N (2020). Omega-3 Polyunsaturated Fatty Acids EPA and DHA as an Adjunct to Non-Surgical Treatment of Periodontitis: a Randomized Clinical Trial. Nutrients.

[28] Castro dos Santos NC, Andere NB, Araujo CF, de Marco AC, Kantarci A, Van Dyke TE (2020). Omega-3 PUFA and aspirin as adjuncts to periodontal debridement in patients with periodontitis and type 2 diabetes mellitus: Randomized clinical trial. J Periodontol.

[29] Deore GD, Gurav AN, Patil R, Shete AR, Naiktari RS, Inamdar SP (2014). Omega 3 fatty acids as a host modulator in chronic periodontitis patients: a randomised, double-blind, palcebo-controlled, clinical trial. J Periodontal Implant Sci.

[30] Graves DT, Cochran D (2003). The contribution of interleukin-1 and tumor necrosis factor to periodontal tissue destruction. J Periodontol.

[31] Pan W, Wang Q, Chen Q (2019). The cytokine network involved in the host immune response to periodontitis. Int J Oral Sci.

[32] Panezai J, van Dyke T (2023). Polyunsaturated Fatty Acids and Their Immunomodulatory Actions in Periodontal Disease. Nutrients.

[33] Zarrough AE, Hasturk H, Stephens DN, Van Dyke TE, Kantarci A (2023). Resolvin D1 modulates periodontal ligament fibroblast function. J Periodontol.

[34] Albuquerque-Souza E, Schulte F, Chen T, Hardt M, Hasturk H, Van Dyke TE (2020). Maresin-1 and Resolvin E1 Promote Regenerative Properties of Periodontal Ligament Stem Cells Under Inflammatory Conditions. Front Immunol.

[35] Tsukasaki M (2021). RANKL and osteoimmunology in periodontitis. J Bone Miner Metab.

[36] Baeza M, Garrido M, Hernández-Ríos P, Dezerega A, García-Sesnich J, Strauss F (2016). Diagnostic accuracy for apical and chronic periodontitis biomarkers in gingival crevicular fluid: an exploratory study. J Clin Periodontol.

[37] Rose-John S (2012). IL-6 trans-signaling via the soluble IL-6 receptor: importance for the pro-inflammatory activities of IL-6. Int J Biol Sci.

[38] Faulkner E, Mensah A, Rodgers AM, McMullan LR, Courtenay AJ (2022). The Role of Epigenetic and Biological Biomarkers in the Diagnosis of Periodontal Disease: A Systematic Review Approach. Diagnostics (Basel).

[39] Cafiero C, Spagnuolo G, Marenzi G, Martuscelli R, Colamaio M, Leuci S (2021). Predictive Periodontitis: The Most Promising Salivary Biomarkers for Early Diagnosis of Periodontitis. J Clin Med.

[40] Feres M, Retamal-Valdes B, Faveri M, Duarte P, Shibli J, Soares GM (2020). Proposal of a Clinical Endpoint for Periodontal Trials: The Treat-to-Target Approach. J Int Acad Periodontol.

[41] Khiste SV, Ranganath V, Nichani AS, Rajani V (2011). Critical analysis of biomarkers in the current periodontal practice. J Indian Soc Periodontol.

[42] Nguyen T, Sedghi L, Ganther S, Malone E, Kamarajan P, Kapila YL (2020). Host-microbe interactions: Profiles in the transcriptome, the proteome, and the metabolome. Periodontol 2000.

[43] Feres M, M Duarte P, Figueiredo LC, Gonçalves C, Shibli J, Retamal-Valdes B (2022). Systematic and scoping reviews to assess biological parameters. J Clin Periodontol.

[44] Babu M, Snyder M (2023). Multi-Omics Profiling for Health. Mol Cell Proteomics.

[45] Norris PC, Serhan CN (2018). Metabololipidomic profiling of functional immunoresolvent clusters and eicosanoids in mammalian tissues. Biochem Biophys Res Commun.

[46] Nguyen T, Sedghi L, Ganther S, Malone E, Kamarajan P, Kapila YL (2020). Host-microbe interactions: Profiles in the transcriptome, the proteome, and the metabolome. Periodontol 2000.

[47] Fatima T, Khurshid Z, Rehman A, Imran E, Srivastava KC, Shrivastava D (2021). Gingival Crevicular Fluid (GCF): A Diagnostic Tool for the Detection of Periodontal Health and Diseases. Molecules.

